# Role of Gut Microbiome in Cardiovascular Events: A Systematic Review

**DOI:** 10.7759/cureus.32465

**Published:** 2022-12-13

**Authors:** Naushad M Mansuri, Neelam K Mann, Shariqa Rizwan, Afrah E Mohamed, Ahmed E Elshafey, Akanchha Khadka, Emmanuel Mudika Mosuka, Kalanchige N Thilakarathne, Lubna Mohammed

**Affiliations:** 1 Research, California Institute of Behavioral Neurosciences & Psychology, Fairfield, USA

**Keywords:** bacteria in the intestines, myocardial infarction, atherosclerosis, cardiovascular disease, cardiovascular events, bacteroidetes, firmicutes, gastrointestinal flora, gut microbiota, gut microbiome

## Abstract

The gut microbiome helps maintain homeostasis in the body, but what if the gut experiences imbalance? It would lead to dysbiosis - which is involved in multiple diseases, including but not limited to cardiovascular diseases, the most common cause of mortality around the globe. This research paper aims to explain all the possible mechanisms known linking the gut microbiome to the contribution of worsening cardiovascular events. PubMed and Google Scholar were thoroughly explored to learn the role of the gut microbiome in cardiovascular events. A systematic review was performed using the Preferred Reporting Items for Systematic Reviews and Meta-Analyses (PRISMA) guidelines to analyze the possible pathways and the metabolites included in the study. Thirteen review articles were selected based on the assessment of multiple systematic reviews (AMSTAR) and the scale for the assessment of non-systematic review articles (SANRA) checklist scores. In this article, we have discussed the role of the gut microbiome in atherosclerosis, hypertension, metabolic disorders such as diabetes and obesity, coronary artery disease, etc. Various pathways to modify the gut microbiome are also discussed, along with the use of probiotics. Finally, we discussed the role of trimethylamine N-oxide (TMAO), a gut microbiome metabolite, as a biomarker for the prognosis of various diseases. This study concluded that the gut microbiome does play a crucial role in the worsening of cardiovascular diseases and the metabolites of which can be used as biomarkers in the prognosis of cardiovascular events.

## Introduction and background

As the famous quote goes, “All diseases begin in the gut” quoted by the famous Hippocrates, also known as the Father of Medicine. The gut microbiome plays a central part in maintaining the physiology of the human body, the evolution of the immune system, and providing the body with nutrients necessary for growth and development [1]. Each individual has a different gut microbial composition that varies drastically, and multiple factors play their part in maintaining the homeostasis of the gut microbiome [2]. However, gut microbial colonies are primarily composed of essential bacteria such as *Bacteroides, Firmicutes, Acinetobacteria, Proteobacteria, and Cerucomicrobia phyla*, over 90% of which are composed of *Bacteriodes and Firmicutes* [2].

Multiple factors affect the gut microbiota composition, such as eating habits, environmental factors, genetic factors, age, use of antibiotics, etc. [3]. Therefore, maintaining homeostasis among the gut microbial colonies is vital for human health, and any disruption in the gut microbiome, also referred to as dysbiosis, can activate or accelerate any number of diseases, including but not limited to cardiovascular diseases (CVD) [3].

According to the Centers for Disease Control and Prevention, CVD are the leading cause of mortality in the United States, causing about 25% of deaths [4]. Diet has long been thought to play a role in developing CVD [5]. Other known risk factors associated with CVD are smoking, obesity, dyslipidemia, hypertension, and physical inactivity [6]. The gut microbiome is now described as an organ, and its effect on atherosclerosis and hypertension is now well established. Recent studies have consistently shown that specific gut metabolites characteristics have been related to coronary artery disease (CAD) and heart failures, such as a decrease in the composition of butyrate-producing gut microbiota and an increase in trimethylamine-N-oxide (TMAO) metabolites produced by the gut microbiome [7].

One of the famous metabolites of fatty foods, TMAO, is formed after the breakdown of choline, phosphatidylcholine, L-carnitine, and other trimethylamine (TMA) containing nutrients by TMA lyase is now known to be related to thrombosis and atherosclerosis, and other cardiometabolic diseases such as diabetes, obesity, etc. [5,8,9]. Furthermore, short-chain fatty acids (SCFA) products such as acetate, propionate, and butyrate, formed in the gut by fermentation of non-digestible carbohydrates, are related to blood pressure regulation, metabolic regulation, and the gut barrier function [9].

This review will summarize the known mechanisms relating the gut microbiome to cardiovascular events such as atherosclerosis, hypertension, CAD, and other cardiometabolic disorders. We will also discuss whether modifying the gut microbiota with drugs can improve the outcome for patients with CVD. Finally, we will also explore the potential of using the enzymes secreted by the gut microbiome as biomarkers in the evolution of cardiovascular events.

## Review

Methods

Protocol

This systematic review followed the latest Preferred Reporting Items for Systematic reviews and Meta-Analysis (PRISMA) guidelines [10].

Search Strategy

For this systematic review, two databases PubMed and Google Scholar were explored thoroughly, employing the appropriate keywords and medical subject headings (MeSH) using the Boolean, “AND, OR and NOT” technique. The following keywords were used: (1) Gut microbiome OR Gut Microbiota OR Gastrointestinal flora OR Bacteria in the GI tract OR Bacteria in the intestines OR Bacteroidetes OR Firmicutes, (2) Cardiovascular Events OR Cardiovascular Diseases OR Myocardial Infarction OR Myocardial Ischemic Events OR Atherosclerosis.

The MeSH strategy applied to PubMed was (Gut microbiome OR Gut Microbiota OR Gastrointestinal flora OR Bacteria in the GI tract OR Bacteria in the intestines OR Bacteroidetes OR Firmicutes OR ("Gastrointestinal Microbiome/drug effects" (Majr) OR "Gastrointestinal Microbiome/etiology" (Majr) OR "Gastrointestinal Microbiome/genetics" (Majr) OR "Gastrointestinal Microbiome/immunology" (Majr) OR "Gastrointestinal Microbiome/physiology" (Majr)) AND Cardiovascular Events OR Cardiovascular Diseases OR Myocardial Infarction OR Myocardial Ischemic Events OR Atherosclerosis OR ("Cardiovascular Diseases/abnormalities" (Mesh) OR "Cardiovascular Diseases/adverse effects" (Mesh) OR "Cardiovascular Diseases/anatomy and histology" (Mesh) OR "Cardiovascular Diseases/blood supply" (Mesh) OR "Cardiovascular Diseases/complications" (Mesh) OR "Cardiovascular Diseases/cytology" (Mesh) OR "Cardiovascular Diseases/diagnosis" (Mesh) OR "Cardiovascular Diseases/drug effects" (Mesh) OR "Cardiovascular Diseases/history" (Mesh) OR "Cardiovascular Diseases/immunology" (Mesh) OR "Cardiovascular Diseases/metabolism" (Mesh) OR "Cardiovascular Diseases/microbiology" (Mesh) OR "Cardiovascular Diseases/pathology" (Mesh) OR "Cardiovascular Diseases/pharmacology" (Mesh) OR "Cardiovascular Diseases/physiology" (Mesh) OR "Cardiovascular Diseases/physiopathology" (Mesh) OR "Cardiovascular Diseases/psychology" (Mesh) OR "Cardiovascular Diseases/rehabilitation" (Mesh) OR "Cardiovascular Diseases/surgery" (Mesh) OR "Cardiovascular Diseases/therapy" (Mesh)).

Inclusion/Exclusion Criteria

This study has restricted its search to only traditional and systematic reviews published in the last five years, i.e., 2017-2021, till the 15th of August 2021. We have only included the papers written in the English language and available free full-text articles. This review has excluded all studies that were performed other than in humans. Randomized control trials and meta-analysis studies were also excluded. Gray literature was also excluded from the study.

Assessment of Study Quality

All the traditional or narrative reviews were assessed using a scale for the quality assessment of narrative review articles (SANRA) checklist, and all the systematic reviews were assessed using the assessment of multiple systematic reviews II (AMSTAR II) tool. Sixty-seven full-text articles were assessed and scored from 0-12 for SANRA and 0-16 for systematic reviews. The studies that scored more than 10 for SANRA and more than 14 for AMSTAR II were marked eligible for data extraction.

Results

Study Selection

**Figure 1:**
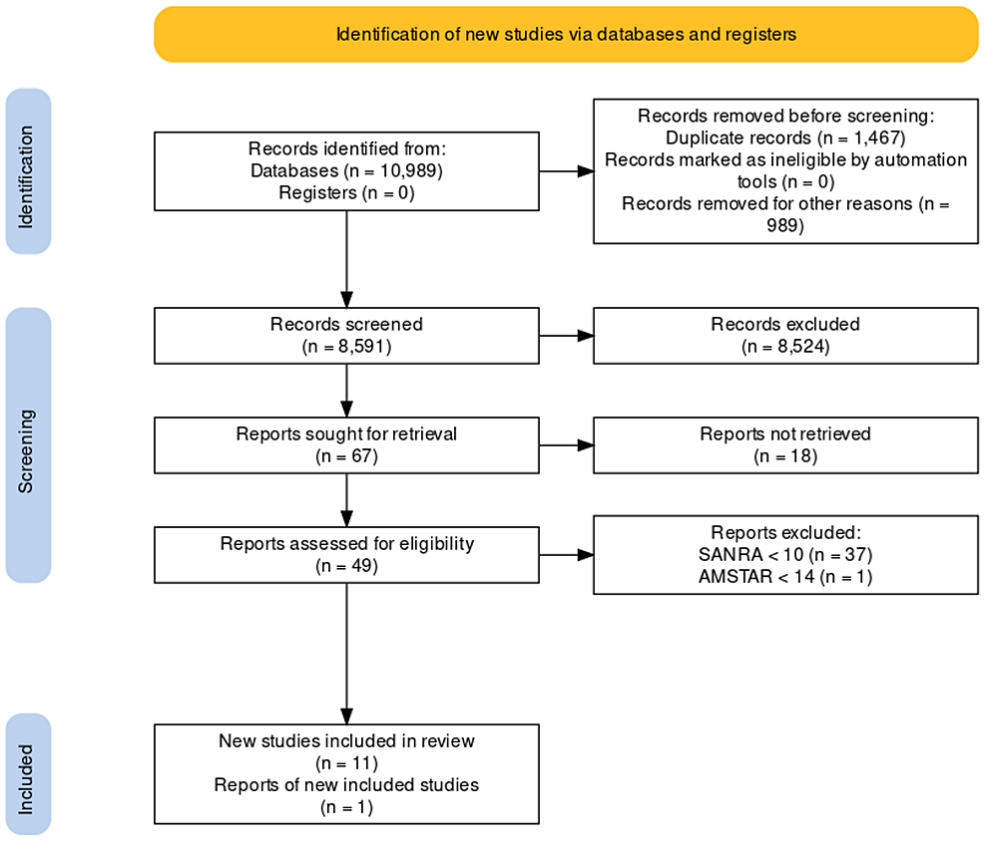
PRISMA flow diagram (downloaded from PRISMA Flow Diagram). SANRA - Scale for the Assessment of Narrative Review Articles, AMSTAR - Assessment of multiple systematic reviews

A total of 2,472,423 were found; after applying the inclusion-exclusion criteria, 10,989 results remained, of which 10,000 references were downloaded from PubMed and 58 references were downloaded from Google Scholar. All the 10,058 references were then transferred to Microsoft Excel, and 1467 duplicate articles were removed manually, screening them in Microsoft Excel. After removing the duplicates, all the 8591 articles were thoroughly screened for titles to avoid losing pertinent information. A total of 8524 articles were removed after screening the titles and abstracts, and only 67 studies were sought for retrieval. All 67 articles were rigorously studied, and 49 of those articles were assessed for eligibility. Eleven of those articles were marked as eligible based on the SANRA score of 10 or more for traditional reviews and the AMSTAR II score of 14 or more for systematic reviews, as mentioned above. One additional systematic review was added from PubMed. Details are described in the PRISMA flow chart (Figure 1).

Data Extraction

Data selection was done autonomously by the first author, NM, and was reviewed by the first and second authors, NM and NM. Data extraction was done autonomously by both the first and second authors, and the disagreements were resolved using a mutual discussion. If still needed, the third and fourth review authors settled the differences.

Table 1 represents the most crucial findings from the studies selected.

**Table 1:** A tabular summary of all the studies included in this systematic review. TLR - toll-like receptor, HTN - hypertension, TMAO - trimethylamine N-oxide, NF-KB - nuclear factor K beta, IRF3 - Interferon regulatory factor 3, CAD - coronary artery disease, TNF - tumor necrosis factor, SCFA - short chain fatty acids, GPR - G protein-coupled receptor, CVDs - cardiovascular diseases, GPR43 - G protein-coupled receptor 43, FFAR2 - free fatty acid receptor 2, GPR41 - G protein-coupled receptor 41, FFAR3 - free fatty acid receptor 3, LDL - low-density lipoprotein, BMI - body mass index, MI - myocardial infarction, GRACE - global registry of coronary events, BNP - brain natriuretic peptide, GFR - glomerular filtration rate, DMB - dimethyl butanol, DOCA - deoxycorticosterone acetate, ACE - angiotensin-converting enzyme, MyD88 - myeloid differentiation factor 88, TMA - trimethylamine, vWF - von Willebrand factor, Olfr78 - Olfactory receptor-78

Author	Location	Study Type	Relationship to Gut Microbiome	Main Findings
Alina et al. 2021 [11]	United Kingdom	Traditional Review	HTN	1. Dysbiosis has an HTN effect on the host. 2. Two pathways were discussed: 2.1 - Lipopolysaccharide activates TLR 4, starting multiple integrated pathways leading to HTN. 2.2 - Lipopolysaccharide mediates inflammation leading to endothelial dysfunction and HTN. 3. Probiotics decrease Blood Pressure by reducing vascular inflammation and improving vasorelaxation.
Yee Teng et al. 2021 [12]	Malaysia	Systematic Review	Stroke outcomes	1. *FRIMICUTES *is the most critical phylum associated with ischemic stroke. 2. TMAO enhances atherosclerosis which leads to strokes and other cardiovascular diseases. 3. Higher TMAO levels were associated with ischemic strokes on admission before a significant decrease at 48 hrs. 4. Elevated *Firmicutes/Bacteroides* ratio is the hallmark of aging. 5. Butyrate is a metabolite produced by *Odoribacter* that enhances the anti-inflammatory effect by lessening the Lipopolysaccharide-induced NF-KB activation, resulting in decreased ischemic injury by increasing IRF3 activity.
Andrea et al. 2021 [13]	Italy	Traditional Review	CAD	1. TMAO exerts dyslipidemic, pro-inflammatory, and pro-thrombotic effects. 2. Decrease in overall growth microbial richness and diversity, e.g., reduction in *Faecalibacterium, Oscillibacter, Rosburia, Bifidobacterium, Coprococcus, and Butyvibrio* and an increment in the growth of* Prevotella and Klebsiella *is associated with idiopathic HTN, which is also one of the most critical CAD risk factors. 3. In-CAD – An increase in *Firmicutes and Fusobacteria* was noticed, while a decrease in *Bacteroidetes and Proteobacteria *was observed. 4. TMAO positively correlates to metabolic syndrome, total cholesterol, low-density lipoprotein, apolipoprotein levels, and TNF alpha levels. 5. SCFA bind to GPR and play an immunomodulatory and anti-inflammatory role in the human body. 5. SCFA has also been shown to protect against diet-induced obesity, improve lipid metabolism and glucose control via bonds with GPR and Free Fatty Acid receptors - GPR43/FFAR2 and GPR41/FFAR3. 6. Probiotics can improve LDL cholesterol, blood pressure, inflammatory mediators, blood glucose levels, and BMI.
Xinyu et al. 2020 [14]	Republic of China	Systematic Review	TMAO to HTN	1. People with high circulating TMAO concentration have a 12% increased risk of HTN than people with low circulating TMAO. 2. TMAO is not only affected by gut microbiota but is also influenced by diet.
Chonyang L e al. 2018 [15]	USA	Traditional Review	Cardiovascular diseases	1. Of all the metabolites of Phosphatidylcholine, e.g., Choline, Betaine, and TMAO, TMAO demonstrated the most vital link to CVD. 2. TMAO is linked to atherosclerosis accelerating the process and associated with major cardiovascular events such as MI, stroke, and death. 3. Poor prognosis is associated with TMAO at years post-MI compared to GRACE risk score or other biomarkers for CAD such as copeptin and proenkephalin, mild regional pro adrenomedullin, and pro-substance. 4. Increased TMAO levels are also a prognostic factor for Systolic Heart Failure, e.g., TMAO levels directly correspond to BNP and inversely correspond to estimated GFR. 5. The exact pathophysiology associated with TMAO remains to be explained.
Stefanie et al. 2018 [16]	Germany	Traditional Review	Cardiovascular and cerebrovascular diseases	1. In early life, temporary treatment with low-dose antibiotics is enough to induce changes in the constitution of the gut microbiome, increasing the obesity associated with diet. 2. A vascular firewall is represented by the venous blood in the small intestine that traps the metabolites derived from the gut microbiome, including molecular patterns, and prevents it from entering the systemic circulation. 3. TLR/MyD88 (myeloid differentiation 88) pathways promote neutrophil aging through signals from gut microbiota; also, in a hyperlipidemic state, deficiency of TLR adaptor Myd88 decreased early atherosclerosis by reducing recruitment of macrophages. 4. Increased production of SCFAs is associated with a high-fiber diet. SCFAs can help prevent HTN and Heart failure through acetate that can potentially downregulate the renin-angiotensin-aldosterone system. 5. Excess salt intake depletes* Lactobacillus murinus,* which is associated with increased Blood Pressure. 6. Increased levels of periodontal organisms like *Aggregatiacter actinomycetemcomitans* in the saliva are associated with high CAD risk. 7. TLR expression on vascular endothelium may predispose to atherosclerosis and is a critical pathway enhancing the formation of cerebrovascular malformations leading to strokes. 8. In the liver endothelium, the gut microbiome functions as an activator for TLR 2, which regulates vWF synthesis and vWF plasma levels. 9. Probiotics have the potential to become an excellent therapeutic choice for preventing cardiovascular disease.
Edward S et al. 2018 [9]	Published online	Traditional Review	Metabolic and cardiovascular health	1. SCFAs have various local effects in the gut, including splanchnic and peripheral tissues, all of which appear to improve metabolic function and have direct and indirect impacts on CVD risk markers. 2. SCFA is essential for maintaining a healthy gut. SCFA also plays a central role in maintaining epithelial integrity through tight junction protein coordination that controls the path between the portal system and the gut lumen. 3. Three main SCFA - (I) Acetate, (II) Propionate, (III) Butyrate. 4. Acetate and Propionate – are involved in the production of renin regulated by Olfr78 and counter-regulated by FFAR3 whereas, Butyrate - attenuates ACE II-induced, renal prorenin receptors, and renin expression.
Wilson W H et al. 2018 [17]	USA	Traditional Review	Cardiovascular health and disease	1. Dietary intervention to alter gut microbiota composition could be a promising therapeutic target. 2. Choline structural analog 1,3 DMB that inhibits TMA lyase can treat cardiometabolic phenotypes in human subjects. 3. *Lactobacillus spp*. enhances insulin secretion, promotes incretin secretion in obese glucose-tolerant subjects, and reduces toxin production such as dimethylamine and nitroso-dimethylamine in patients with chronic kidney disease. 4. Engineered probiotics with enhanced effectiveness of the beneficial effects, e.g., N-acyl phosphatidylethanolamines expressing* E. Coli Nissle 1917*, reduce obesity induced by a high-fat diet, insulin resistance, and hepatosteatosis in mice. 5. Individualized treatment plans depending on the microbiome can provide potential treatment plans for cardiometabolic disorders. 6. All TMAO-associated metabolites—choline, betaine, and L-carnitine—had a positive linkage with prevalent CVDs and incident cardiovascular events in the original human trials of more than 1,800 stable cardiac people undergoing elective coronary angiography. 7. In individuals with acute coronary syndrome, circulating TMAO was linked to the presence of susceptible coronary plaque, plaque rupture, and long-term risks of incident cardiovascular events.
Sarah et al. 2017 [18]	USA	Traditional Review	HTN	1. There are two genomes in the human body, (I) the Human or Host genome that is rigid and studied thoroughly, (II) the microbiome or the SECOND genome that can be modified by nutrition and transplantation. 2. The second genome can be a potential treatment for obesity and HTN. Increased *Firmicutes* to* Bacteroidetes *ratio is involved in metabolic disease. High fructose and salt disturb the gut microbiome. 3. Gut microbiome modification can be a potential treatment for obesity and HTN.
Yoriko et al. 2017 [19]	USA	Systematic Review	CVDs	1. Regardless of traditional risk factors, higher levels of TMAO are linked to an increase in major adverse cardiovascular events and death. 2. Intestinal bacteria produce TMA from dietary choline and L-carnitine, which are subsequently absorbed into the bloodstream and oxidized to TMAO by the liver enzyme flavin monooxygenase 3. 3. In vivo, TMAO interacts directly with platelets, modifying calcium transmission and inducing platelet hyperactivity and a prothrombotic phenotype.
Qi et al. 2017 [20]	USA	Traditional Review	HTN	1. HTN is related to a greater *Firmicutes* to *Bacteroidetes* ratio. 2. A decrease in Acetate and Butyrate-producing bacteria and an increase in lactate-producing bacteria are associated with HTN. 3. In DOCA -salt mice, a high-fiber diet coupled with acetate supplementation rectifies gut dysbiosis and is associated with decreased blood pressure. 4. Like in rats, subjects with HTN or pre-HTN demonstrate similar characteristic changes in the gut microbiome composition. 5. Pre- and probiotics reduce blood pressure and the risk of HTN via modifying cholesterol, inflammation, blood glucose levels, and the renin-angiotensin system.
Yuichiro et al. 2017 [21]	Japan	Traditional Review	General health and disease	1. *Lactobacilli* were found in more significant numbers in diabetes patients' feces, while *Clostridium coccoides, Atopobium clusters, and Prevotella* were found in lower numbers. 2. Stroke patients had significantly higher *Lactobacillus ruminis *bacterial counts than controls. According to the multivariable analysis, regardless of age, HTN, or type 2 diabetes, increased bacterial counts of the *Atopobium cluster and L. ruminis *and decreased counts of the *Lactobacillus sakei* subgroup are associated with increased ischemic stroke risk.

Discussion

This section will discuss the gut microbiome's role in disease processes associated with cardiovascular events like atherosclerosis, hypertension (HTN), and obesity. First, we will review the mechanism by which the gut microbiome affects several immunological pathways and how it can trigger inflammation and increased risk of thrombosis that can lead to strokes. We will also discuss the potential of modifying the gut microbiome to improve the outcomes for CVDs and, finally, the use of gut microbiome metabolites as biomarkers for CVDs.

Gut Microbiome and Risk of Atherosclerosis

Atherosclerosis is an immunoinflammatory disease that causes blockages in the large and medium arteries, resulting in acute CVD, and is a significant cause of mortality [22,23]. In addition, cholesterol and fatty acids, fibrotic substances, macrophages, dendritic cells, and other host immune cells are present in atherosclerotic plaques [23,24]. Thus, endothelial dysfunction, inflammation, and elevated low-density lipoprotein (LDL) levels are all factors that lead to atherosclerosis [22-24].

Plasma lipopolysaccharides concentration leads to vascular endothelial dysfunction by nicotinamide adenine dinucleotide phosphate (NADPH) oxidase pathway mediated by toll-like receptor-4 (TLR4) by generating reactive oxygen species (ROS) and following endothelial nitric oxide synthase (eNOS) deactivation and decreased endothelial nitric oxide (NO) bioavailability leading to endothelial dysfunction [11].

When the gut microbiome is disturbed by any number of reasons, including but not limited to environmental or dietary stress, also called "DYSBIOSIS" [24]. Dysbiosis can then trigger inflammatory process through various pathways such as the overproduction of pro-atherogenic factors like C-reactive protein, interleukin 18, interleukin 1, interleukin 16, and tumor necrosis factor (TNF), as well as increased expression of adhesion molecules like vascular endothelial adhesion molecule 1 (VCAM-1), production and release of perivascular derived adipocytes, ROS, and hormones like corticosteroids and sex hormones, all have a nonspecific inflammatory role enhanced by dysbiosis. It also has a direct cytokine-related influence on the autonomic nervous system [25,26]. In addition, when hormones start talking to the immune system, it is called neuroendocrine-immune crosstalk, which has a vital role in the inflammatory process. When this happens, the homeostasis gets disturbed and leads to altered levels of LDL cholesterol, plasma triglycerides, and high-density lipoprotein (HDL) cholesterol, leading to an increase in the oxidation of LDL cholesterol, enhancing plaque formation and instability [25].

According to several studies, hypercholesterolemia has also been linked to gut microbiome products such as TMAO, SCFA, and bile acids (BAs). They play a vital role in maintaining cardiovascular health, and any dysregulation can potentially lead to increased atherosclerotic risk [24-27].

BAs are the principal means of cholesterol removal from the body. Now a high efficiency of cholesterol to coprostanol metabolism was suggested to reduce cardiovascular risk, which is carried out by *Lactobacillus and Eubacterium coprostanoligenes*, the enzymes for which are unknown [27]. Certain bacteria in the small intestine, especially gram-positive ones such as *Lactobacillus, Clostridia, Listeria, and Bifidobacteria,* have bile salt hydroxylase capable of deconjugating (i.e., remove glycine or taurine) that prevent the BA absorption [27]. Free glycine or taurine is then taken up by the intestine, returned to the liver, and transferred to unconjugated BAs that are not readily absorbed [27].

Choline, phosphatidylcholine, and L carnitine metabolize to TMA, which is oxidized into TMAO by flavin monooxygenase-3 in the liver [25,27,28]. TMAO levels are set by factors such as genetic variations, diet, and the gut microbiome. Dysbiosis is directly associated with increased TMAO levels, and elevated TMAO levels are associated with poorer outcomes with CAD and chronic heart failure [27,28]. In addition, high TMAO levels cause an increase in the expression of cluster of differentiation 36 (CD36) and scavenger receptor A (SRA) on macrophages involved in the uptake of oxidized LDL, also increase nuclear farnesoid X receptor (FXR) and small heterodimer partner (SHP) receptor expression, which reduces the reverse cholesterol transport [27]. This increases the formation of foam cells and is a critical step in atherosclerosis [27].

The most crucial bacterias for producing SCFA are the clostridial clusters IV and XIVa of* firmicutes*, including *Eubacterium, Roseburia, Faecalibacterium, and Coprococcus* [27]. SCFA reduce cholesterol levels in the blood by upregulation of sterol-regulatory element-binding protein 2 (SREBP-2), LDL receptor, and cholesterol 7 α-hydroxylase (CYP7A1) genes in the liver by increasing the liver cholesterol uptake and excretion of BA in the feces [27]. In the small intestine, butyrate is used as the energy source, and acetate and propionate are absorbed, hence, the concentration of butyrate is much lower in the plasma than acetate and propionate [29]. Butyrate also inhibits histone deacetylase (HDAC), decreases the synthesis of TNF α, interleukin 12, and interferon γ (IF-γ), and increases the synthesis of anti-inflammatory cytokines such as interleukin 10 by monocytes [29].

**Figure 2:**
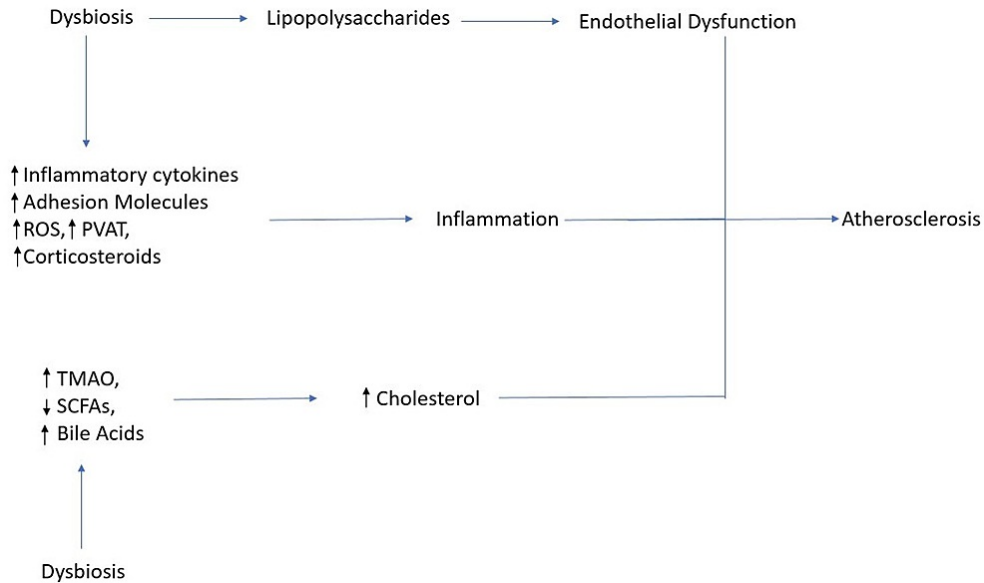
Explains the pathways by which the gut microbiome can enhance the process of atherosclerosis. ROS - reactive oxygen species, PVAT - perivascular derived adipocytes, TMAO - trimethylamine N-oxide, SCFA - short chain fatty acids

Dysbiosis leads to an imbalance in any of these processes and can potentially lead to atherosclerosis as described in Figure 2.

Effect of the Gut Microbiome on Hypertension

HTN defined by SBP > 140 and DBP > 90, affects over 1.2 billion people all over the globe [30]. According to the new American College of Cardiology, the cut-off to HTN has been raised to > 130/80 [31]. It is also linearly associated with obesity and the risk of cardiovascular events. Multiple factors such as age, genetics, stress, BMI, and sodium and potassium intake play a significant role in the pathology of HTN [30]. Nearly 10% of the cases are of secondary HTN, i.e., the cause of which is known, but it is still unknown for 90% of patients [18].

Recent studies have shown that the gut microbiome also plays a significant role in the pathogenesis of HTN, the theories of which will be discussed in this section. For example, spontaneously hypertensive rats infused with angiotensin II and human hypertensive patients show similar gut dysbiosis characterizing an increase in* Firmicutes to Bacteroides* ratio [18,20,31,32]. Mainly three hypotheses were discussed, which are as follows:

One hypothesis suggests that gut dysbiosis triggers the loss of SCFA-producing bacteria, leading to decreased butyrate, propionate, and acetate. SCFA provides energy and makes the intestinal epithelium stronger [31,33]. A decrease in SCFA also plays an inflammatory role in the body by decreasing the production of butyrate leading to inflammation and renal interstitial injury leading to elevated salt and water reabsorption. Reduced output of propionate reduces the expression of G-protein coupled receptor 41, leading to decreased vasodilation. Moreover, a reduced acetate leads to increased olfactory receptor 78 (Olfr78), causing a surge in renin and leading to HTN [31,33,34].

Another hypothesis includes lipopolysaccharides that cross the weakened intestinal barrier caused by dysbiosis 11. Thus, two theories have been proposed that link HTN to lipopolysaccharides. One includes endothelial dysfunction the other one comprises vascular inflammation [11].

Lipopolysaccharides causing endothelial dysfunction - lipopolysaccharides exposure leads to activation of TLR4 mediated oxidative pathway and generation of reactive oxygen species through NADPH oxidase and following eNOS deactivation and decreased endothelial nitrous oxide bioavailability leading to endothelial dysfunction [11].

Lipopolysaccharides causing vascular inflammation - lipopolysaccharides exposure leads to TLR4-mediated inflammatory pathway activation. It further leads to p38MAPK (mitogen-activated protein kinase) activation, causing nuclear factor kappa beta inhibitor (IκBα) protein degradation and following p65 NF-κB translocation to the nucleus. Translocation of nuclear factor-kappa B (NF-κB) leads to increased production of IL-6 and adhesion molecules such as intercellular adhesion molecule-1 (ICAM-1), VCAM-1, and E-selectin [11].

**Figure 3:**
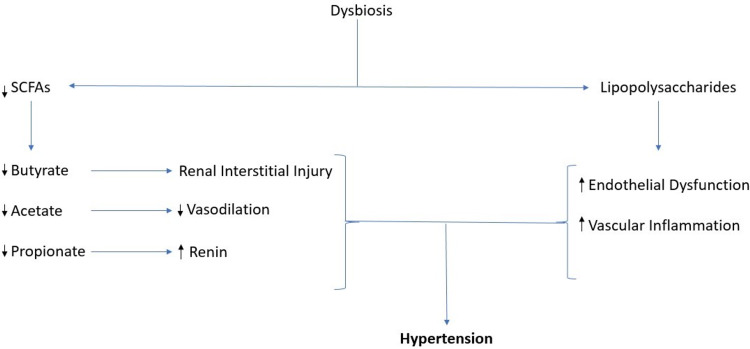
Shows the pathways associated with dysbiosis and hypertension. SCFA - short chain fatty acids

Both theories mentioned above increase the risk of HTN 11, which is explained in Figure 3.

Sarah et al. suggest another theory that involves genetics as its basis [18]. It is now established that genetic and environmental factors cause HTN, but it is not precisely known which genome is associated with HTN [18]. Recent evidence shows that, unlike the rigid host genome, the modifiable genome of the gut microbiome also has a significant effect on blood pressure [18]. Unfortunately, it is very minutely studied and has little evidence to follow [18].

Effect of the Gut Microbiome on CVDs and CADs

Gut microbiomes control the nutrient uptake and its processing, help maintain the gut barrier, prevent pathogenic bacteria's passage into the host body, and stimulate the host immune system to defend against pathogens [17]. Dysbiosis is linked to the advancement of various CVDs such as diabetes mellitus, CAD, and obesity [17,35,36].

The gut microbial species such as *Akkermansia, Bifidobacterium, Bacteroides, Faecalibacterium, and Rosburia* were found to have a protective role in glucose metabolism and were less abundant in cases with type II diabetes mellitus. In contrast, *Firmicutes, Ruminococcus, Fusobacterium, and Blautia* were more productive [35]. Subjects with type II DM also had decreased levels of SCFA, especially butyrate-producing bacteria [17,35,37].

Obesity is another chronic disease with significant concern for high cardiovascular event risk. Dysbiosis can trigger obesity by interfering with the host metabolism and energy homeostasis [38]. In addition, a high-fat diet affects the gut microbiota as it decreases the levels of* Bifidobacterium, Tenericutes, phylum Bacteroidetes*,* Bacteroides, Lactobacillus, Roseburia, Eubacterium rectale, and Blautia coccoides* and increases the abundance of *Firmicutes, Acinetobacteria, and Proteobacteria, Deltaproteobacteria, Gammaproteobacteria* and pathobionts such as *Staphylococcus spp., Odoribacter spp., Neisseria spp., and Propionibacterium spp.* [38]. Overall, obese cases with metabolic disorders have less microbiome diversity than subjects who were obese but do not have metabolic disorders. Weight loss increases the portion of *Bacteroidetes*, and high fiber and a low-fat diet are associated with lowering the levels of *Firmicutes *[38].

Several risk factors have been associated with CAD, such as obesity, diabetes, hypercholesterolemia, etc. [13]. A close association has been found linking CAD and the gut microbiome [13]. TMAO is one of the most investigated pathways for linking gut microbiota and CAD [13]. The mechanism is unknown, but it is established that TMAO directly correlates with CAD and gut microbiome [13].

Modification of Gut Microbiome to Improve the Outcomes for Cardiovascular Events

Modification of the gut microbiome can be damaging when the gut microbiome undergoes atypical changes, as the gut microbiome plays a significant role in various bodily functions, including nutrient absorption, vitamin synthesis, and the promotion of angiogenesis [39]. The gut microbiota contains primarily three phyla of bacterial species, i.e., *Bacteroidetes, Firmicutes, and Acinetobacter.* In addition, *E. coli, Lactobacilli, and Streptococci* are discovered in small amounts [39]. Alteration in human gut microbial composition can lead to several diseases [2,24,39-42], but the therapeutic interventions by probiotics, fecal transplantation, and TMA lyase inhibitors have shown promising results [38,39,43].

TMA lyase inhibitors - CVD is associated with elevated TMAO levels, and blocking TMA lyase activity by DMB (3,3-dimethyl-1-butanol, DMB) can prevent the progression of atherosclerosis in mice [43]. Even in humans, DMB was able to suppress TMA production. Further human trials need to be conducted for solid proof [43]. TMAO has been associated with diabetes, chronic kidney disease, atherosclerosis, and other cardiometabolic diseases; therefore, TMA lyase inhibition can prevent these diseases from progressing [43].

Fecal microbial transplant - In experimental models, fecal microbial transplant (FMT) has also been shown to lower insulin resistance and inflammation markers in the host and reverse the elevated insulin levels in the blood [35]. In addition, it also improved pancreatic beta-cell function and lowered the expression of pro-apoptotic molecules such as Bcl-2-associated X protein (BAX) and caspase 3 in the pancreatic beta cells [35].

Probiotics - As a therapeutic intervention for cardiovascular disease, probiotics also improved the atherogenic index, lipid profile and CAD risk index, and serum inflammatory markers [35]. Main probiotic microorganisms include *Lactobacillus rhamnosus, Lactobacillus reuteri, bifidobacteria, Lactobacillus casei, Lactobacillus acidophilus-group, Bacillus coagulans, E Coli strain Nissle 1917, certain enterococci, and a yeast Saccharomyces boulardii* [44]. In addition, human studies with *L. acidophilus L1 *milk showed a significant drop in cholesterol levels [44]. In the future, the recognition of new probiotics and a mixture of probiotics would be vital in preventing multiple CVDs and promoting health in general [39].

Gut Microbiome Metabolites as Biomarkers for Cardiovascular Events and Mortality Risk

TMAO was once considered a waste product of choline metabolism that had no activity in the body, but now there is ample evidence of TMAO being involved in inflammation, CVDs, and obesity [19,45-47]. As previously stated, TMAO is produced by the oxidation of TMA in the gut microbiome [25,27,48]. TMA is absorbed from choline and L-carnitine in the gut by the microbiome, which is then oxidized to TMAO by flavin monooxygenase III [25,27,48]. Saltwater fish has the highest concentrations of TMAO out of all the food sources 48. Red meat, eggs, and dairy products are also rich in TMAO [48].

TMAO was a predictive indicator of prevalent CVDs and future cardiovascular events in several cohort studies [19]. Elevated TMAO levels have been significantly associated with heart failure with diastolic dysfunction and high mortality [48]. In addition, elevated TMAO levels and their precursors are associated with increased mortality risk related to cardiovascular events independent of any other traditional risk factors [19]. TMAO increases proinflammatory cytokines and increases the expression of adhesion molecules, increasing the risk of thrombosis [48].

TMAO also positively correlates with adiposity and metabolic syndrome [47]. TMAO can also be used as an early indicator of non-alcoholic fatty liver disease in cases with no overt symptoms of metabolic syndrome [46,47]. Numerous clinical cohort studies showed that systemic TMAO levels are strongly associated with type II diabetes mellitus [46].

TMAO acts as a biomarker and enhances the early pathological process of atherosclerosis [48]. It is also a renal toxin and works as a prognostic factor for chronic kidney disease patients [48]. TMAO is excreted in the urine and hence significantly affects the glomerular filtration rate [48].

All these critical associations have led researchers to investigate the TMAO blocking agents, which led to promising results in mice, but human trials are yet to be conducted.

Strengths and Limitations

This study has various strengths as it contains the most popular theories hypothesized linking the gut microbiome to cardiovascular events. The studies with a SANRA score of 10 or more and an AMSTAR II score of more than 14 were included. This study also did not have a geographical barrier, as studies from across the globe were included. However, various limitations need to be considered, as this paper included only Literature and Systemic Review Papers and discussed the most general theories regarding the topic. Much of the article is based on pathways involving dysbiosis and its effect on various cardiovascular events, but the limited human trials have limited the hypothesized theories' validity. Many human trials are needed on the genetics involving the gut microbiome, finding unique genetic footprints for various diseases, and validating the ideas explained in the paper. No animal studies were included in this study, and most of the randomized controlled trials were performed on lab rats on this topic. Specific TMAO levels were also not stated, as TMAO levels vary with genetics, population, and the food consumed. Most research articles only focused on the microbiome's bacterial aspect, and much research is needed on viruses, fungi, yeast, etc.

## Conclusions

We summarized most of the theories linking the gut microbiome to atherosclerosis, including endothelial dysfunction, inflammation, and hyperlipidemia, and ideas relating the gut microbiome to HTN and metabolic disorders. This paper explained the detrimental effects of TMAO and the beneficial effects of SCFAs. This study also explained how the body uses lipopolysaccharide as a signal transducer and can trigger inflammation and endothelial dysfunction that contributes to HTN. TMAO is one of the critical metabolites that can be used as a biomarker for cardiovascular event risk and numerous other conditions. The gut microbiome is highly modifiable by multiple mechanisms, including probiotics, fecal microbial transplant, and the use of TMA lyase inhibitor, a newly synthesized molecule. This research is critical because this topic is highly relevant to a large population and is not very well researched. The clinicians also need to be made aware of the growing field and know more in-depth about the pathways included. Future research needs to be based on large populations. People from various parts of the world need to be included in the study to limit bias based on genetic and environmental factors.
